# Community Experiences of Social and Nonmedical Gender-Affirming Care: Interview Study Among Transgender and/or Nonbinary Persons

**DOI:** 10.2196/79179

**Published:** 2026-01-08

**Authors:** Bryah Boutilier

**Affiliations:** 1 School of Health and Human Performance, Dalhousie University, 6283 Alumni Crescent, Halifax, NS, B3H 4R2, Canada, 1 9025777936

**Keywords:** gender-affirming care, social support, health equity, rural health, inclusive health care

## Abstract

**Background:**

Access to care that affirms one’s entire self is essential, especially for gender-diverse individuals. Gender-affirming care includes medical, social, and nonmedical supports to affirm gender identity.

**Objective:**

This study qualitatively examined the importance of social and nonmedical gender-affirming services as described by gender-diverse community members.

**Methods:**

Thematic analysis was conducted on qualitative data from 5 participants (3 rural and 2 urban; 2 with doctoral-level education and 3 health professionals) with experiences accessing gender-affirming care in Nova Scotia, Canada, between October 2023 and November 2023.

**Results:**

Participants included transgender and/or nonbinary individuals who highlighted the significance of social and nonmedical gender-affirming care over traditional medical interventions. Themes included the centrality of belonging, the use of online spaces such as TikTok for gender affirmation, and the emotional impact of barriers such as cost and safety concerns. Four of 5 participants emphasized the importance of social and nonmedical gender-affirming care over medical interventions. Participants stressed the importance of fostering a sense of belonging and accessing supportive communities, which is crucial in navigating transphobic environments without support. Many felt abandoned by public systems and resorted to passing as cisgender due to barriers such as cost in accessing gender-affirming resources. Internet platforms such as TikTok provided valuable guidance, supplementing limited access to medical gender-affirming care. Participants emphasized a crucial need for health care providers to understand basic gender-affirming care, including respect for preferred pronouns and gender identities.

**Conclusions:**

This study found that members of the gender-diverse community significantly value social and nonmedical gender-affirming care services with respect to their well-being. The findings underscore the complex interplay among social support, health care access, and resilience in transgender and/or nonbinary individuals’ lives. This work can aid in exploring how best to educate health care providers in gender-inclusive care and enable increased access to all forms of care that can help affirm an individual’s gender.

## Introduction

Access to care that affirms an individual’s gender identity is fundamental. Gender-affirming care encompasses a wide range of medical, social, and nonmedical services essential for supporting one’s gender identity [1]. These services include hormone therapy, surgeries, mental health support, voice training, and other medical interventions, but they also extend to social and nonmedical supports that are equally crucial, such as gear and feelings of belonging [1,2]. It is important to recognize that what may be considered cosmetic or aesthetic for cisgender individuals can be gender-affirming and vital for gender-diverse individuals [3]. For example, clothing, hairstyling, name changes, and gender marker alterations are all powerful affirmations of identity that often carry profound psychological and social benefits for those within the gender-diverse community.

Despite the critical importance of these services, access to medical gender-affirming care is often limited by systemic barriers such as long wait times, restrictive insurance policies, and a lack of knowledgeable health care providers [1,4-6]. As a result, many gender-diverse individuals turn to social and nonmedical resources to express and validate their identities in everyday life [1]. These forms of care can offer more immediate and accessible means of gender affirmation, helping individuals navigate a society that may not always recognize or understand their needs.

Historically, social and nonmedical gender-affirming services have been underrecognized and undervalued by health care providers and insurance coverages, creating barriers to accessing care [1]. Without these supports, many individuals experience increased risk of mental health challenges, discrimination, and social isolation [7,8].

This study aimed to qualitatively examine the importance of social and nonmedical gender-affirming care services in supporting gender identity as experienced by members of the gender-diverse community. By uplifting the lived experiences of individuals, this research sought to highlight the impact of these services on well-being while advocating for additional inclusive policies recognizing the diverse needs of the gender-diverse community.

## Methods

### Study and Context

In Nova Scotia, Canada, gender-diverse individuals continue to face systemic inequities in health care access [6]. In 2021, the Statistics Canada Census of Population began collecting gender diversity data, revealing that Nova Scotia had the highest proportion of transgender and nonbinary people aged ≥15 years in the country [9]. Despite progress in provincial policies, accessing gender-affirming services such as hormones and surgery remains challenging, particularly outside urban centers. Barriers include not only discrimination and stigma but also a lack of knowledgeable health care providers and logistical hurdles such as lengthy wait times, geographic distance, and financial costs [6]. These challenges are exacerbated for individuals in rural or underserved areas, making the need for alternative support systems such as social and community-based services increasingly critical. This context underscores the importance of understanding how community-based and social supports function as vital sources of affirmation. This study aimed to explore the role of social and nonmedical services in affirming gender identity in a context in which traditional medical gender-affirming care is limited or difficult to access.

### Study Design

This study used qualitative methodologies to gather in-depth insights into the experiences of individuals navigating gender-affirming care. An advisory group composed of representatives from community-based organizations that advocate for gender-diverse individuals was established to provide input throughout the research process. This group played a key role in ensuring that the study’s design, recruitment strategy, and research materials were culturally sensitive and aligned with the needs of the gender-diverse community. The advisory group also assisted in refining the interview questions.

### Data Collection

Five semistructured interviews were conducted using probing techniques in a conversational manner. Participants needed to have accessed gender-affirming care in Nova Scotia within the previous 5 years. Participants also had to be aged >18 years and speak and understand English. Interviews were conducted via Zoom (Zoom Video Communications) to increase accessibility in rural areas. Each interview lasted an average of 55 (SD 14) minutes and was audio recorded and transcribed verbatim. Recruitment took place between August 2023 and November 2023 in the province of Nova Scotia. Participants were recruited through snowball sampling initiated via community organizations that serve gender-diverse individuals. Recruitment notices were shared on organizational social media and on university bulletin boards. Interested individuals contacted the research team directly to ensure confidentiality. Recruitment continued until no new themes emerged from the data. Although the final sample consisted of 5 participants, this number was sufficient given the narrow focus of the study, the relative homogeneity of participant experiences, and the high information power of each interview. Consistent with qualitative guidance, information-rich samples with focused aims may reach saturation with small numbers when participants share similar contextual experiences (eg, access to gender-affirming care within the same province) [10,11]. After the fifth interview, no new codes or themes emerged, and all themes were consistently represented across participants. For these reasons, thematic saturation was considered achieved. In recognition of the potential emotional and psychological impact of discussing sensitive topics, participants were offered the opportunity to have a support person from the gender-diverse community present during the interviews to promote a sense of safety and comfort.

### Data Analysis

Thematic analysis [12] was used for the data, with the NVivo software (Lumivero) facilitating the coding and organization of themes. A codebook was developed through a combination of inductive and deductive coding approaches, ensuring that themes emerged both from the data themselves and from preexisting findings in the literature. Data were analyzed solely by the author. During codebook development, the author sought consultation from 2 qualitative research experts who reviewed a subset of coded excerpts and provided feedback on code clarity, organization, and analytic coherence. Their role was advisory rather than analytic; they did not perform independent coding, conduct parallel analysis, or participate in theme generation. This consultation was used to refine the codebook and strengthen analytic rigor and transparency. Example codes included “community as safety,” “passing for protection,” “provider respect,” and “online peer education.”

### Ethical Considerations

This study received ethics approval from the Dalhousie University Department of Research Ethics Board in Social Sciences in August 2023 (2023-6700). This study adhered to ethical guidelines regarding informed consent, confidentiality, and participant well-being throughout all stages of the research process. All participants provided oral informed consent before taking part, including consent for audio recording and use of anonymized quotes. Participants were fully informed of their rights, including the ability to withdraw from the study at any time without consequence. Data were anonymized and stored on encrypted, password-protected drives accessible only to the research team. Participants did not receive an honorarium for taking part.

### Reflexivity

As a heterosexual, cisgender woman with lived experience in rural communities, the author recognizes the privileges she carries in both her gender identity and sexual orientation, which have largely aligned with societal norms and expectations. Her experiences navigating health and social systems have not been shaped by the same systemic barriers or identity-based marginalization that gender-diverse individuals often face. Growing up in a rural setting, the author has witnessed the limitations of local health care systems, particularly regarding specialized and inclusive services. However, she has not had to seek out gender-affirming care or worry that her identity might be invalidated or misunderstood in those settings. Engaging with this research required continuous reflection on these differences and a commitment to listening deeply and respectfully to participants’ narratives. The author approached this work with humility, striving to center the voices and lived experiences of gender-diverse individuals and avoid imposing assumptions shaped by her own positionality.

## Results

Five participants took part in semistructured individual interviews. Three participants identified as living in a rural area, whereas 2 lived in an urban area. One urban-dwelling participant also had experience accessing services in a rural location. Two participants indicated that they had a doctorate-level education, and 3 participants were trained in health care delivery (or allied health care delivery). All participants identified as transgender and/or nonbinary.

Thematic analysis identified four major themes: (1) belonging as care, (2) barriers and safety in accessing resources, (3) informal digital support networks, and (4) provider competency as affirmation. These themes were consistently represented across participants, with minor variation based on rural vs urban context.

All participants highlighted the significance of social and nonmedical aspects of gender-affirming care in supporting their identity. They discussed the significance of services such as gender-affirming gear, hair alteration, and the ability to access spaces that foster a sense of community. Participants stressed that the sense of belonging outweighs the importance of obtaining medical gender-affirming care. Participants consistently emphasized that a sense of belonging within supportive communities was the foundation of their well-being, often surpassing the importance of medical interventions. For all 5 participants, belonging operated as both a psychological anchor and a survival mechanism in contexts in which formal systems felt unsafe or exclusionary. Rural participants, in particular, described community connection as a form of “protective care” against the social isolation and transphobia of their environments. Urban participants also highlighted belonging but described it more as an avenue for identity expression and solidarity than for safety. Across participants, belonging was experienced as daily, relational affirmation—something that no prescription or surgical procedure could substitute. As one participant explained,

Medical care isn’t going to make my life amazing—it’s finding people that I can connect with that can offer circles of support where we love each other.

Feelings of systemic abandonment were common, with 4 participants describing how limited institutional support forced them to rely on self-devised or peer-provided means of protection. “Passing” as cisgender emerged not as a cosmetic preference but as a vital survival strategy in unsafe public or work environments. Rural participants described the physical act of blending in—through clothing, binding, or padding—as a form of harm reduction in communities where visibility could invite violence or exclusion. One participant explained,

Just having that ability to make my pelvis appear more female, it feels important to me, just from a safety standpoint. Blending in, in environments where you're not necessarily going to be among supportive people, is important.

This underscores how nonmedical gender-affirming gear operates at the intersection of safety, identity, and accessibility. The high cost of such gear further amplified participants’ sense of inequity, reinforcing how financial and systemic barriers shape daily decisions about safety and self-expression. One participant stated,

It’s really hard to explain to a straight person that experience of not having access to trans tape, which is not cheap.

The emotional toll of these compromises—needing to hide to survive—was a recurring theme that participants linked to chronic anxiety and diminished well-being. Participants shared that being unable to access these resources can exacerbate feelings of dysphoria, which in turn can lead to severe mental health challenges, including suicidal ideation:

The amount that helps with your mental health and well-being to prevent anything from suicide to just literally living every day feeling like shit is enormous.

Four of 5 participants, particularly those residing in rural communities or facing long wait times for specialist referrals, described turning to online spaces such as TikTok and Reddit for gender-affirming guidance. These participants noted that virtual communities compensated for geographic isolation and distrust of local health care providers. In contrast, the 2 urban participants, who had intermittent access to affirming clinicians, reported using online resources primarily for emotional validation rather than clinical information. Participants described how online platforms helped them navigate the complexities of their gender identity, find resources, and connect with others facing similar challenges. Many found that social media platforms such as TikTok played a key role in their journey. One participant shared how TikTok guided them in using nonmedical transgender tape for the first time, providing tutorials and tips that were crucial for them:

I just immediately went to TikTok and there’s videos with tutorials with different people with different sizes and just like, here’s some tips and tricks [...] I know it’s good to know because I wish someone would have told me this earlier.

This informal support from the internet was often more useful than professional care. One participant explained how they found more helpful information about speech therapy online than from their private therapist:

I found out more [speech therapy techniques] doing research on the internet and following people... That was a much more useful resource for me.

All participants emphasized that provider competency extended beyond medical expertise to include relational respect and cultural humility. While none expected every clinician to specialize in transgender health, participants unanimously stated that the absence of basic gender awareness—such as using correct pronouns or avoiding invasive curiosity—communicated a deeper message of disregard. For some, these encounters shaped whether they would seek care again. Two participants described leaving appointments feeling “invisible” or “unsafe,” whereas others recounted moments of relief when a provider simply affirmed their identity without hesitation. This theme reveals that, for gender-diverse individuals, the therapeutic value of health care interactions often lies not in clinical outcomes but in the acknowledgment of personhood. Inadequate provider training was seen as a systemic issue that perpetuates mistrust and withdrawal from formal care systems, further driving reliance on informal networks.

## Discussion

### Principal Findings

This study reviewed the significant role of social and nonmedical gender-affirming care services in supporting the gender identity of members of the gender-diverse community. The findings suggest that informal and digital networks are not merely substitutes for unavailable formal medical services but also function as parallel systems of affirmation. Participants sought information online not only because of geographic or financial barriers but because these spaces offered identity-affirming interactions often missing in clinical encounters. Thus, the internet served as a trusted site for peer-led knowledge exchange where the authenticity and relatability of the advice outweighed formal credentialing. This illustrates how mistrust in institutional systems drives the creation of community-based health ecologies that are adaptive, resilient, and grounded in lived experience. The main findings reveal that participants highly valued social and nonmedical aspects of gender-affirming care such as gender-affirming gear, hair services, and supportive communities. These findings highlight the importance of fostering a sense of belonging and supportive communities, which are crucial in navigating transphobic environments, particularly in rural settings with limited access to traditional medical gender-affirming care. This aligns with previous research showing that social support is a critical factor in the mental health and well-being of members of the gender-diverse community. For instance, Glynn et al [13] demonstrated that social connections are protective against psychological distress. Additionally, Durwood et al [14] highlighted that social support from family and friends is associated with lower rates of internalizing symptoms such as depression and anxiety among transgender youth.

Participants in our study reported the need to build connections with other gender-diverse individuals who are going through similar experiences. Supportive networks were perceived as essential in mitigating the failures of public systems, particularly in rural areas where access to gender-affirming services is sparse. These findings align with the literature [15-17] and the gender affirmation framework [13], which posits that strong support systems can foster resilience when faced with social oppression. Participants in this study noted that being in a space in which they felt affirmed in their gender identity led to a reduced risk of experiencing suicidal ideation, illustrating the profound psychological impact of social affirmation.

Furthermore, this study sheds light on the innovative strategies used by gender-diverse individuals to access nonmedical gender-affirming care, particularly through the internet. The use of internet platforms such as TikTok was frequently mentioned as a means of seeking guidance and support, filling the gap created by the unavailability of traditional medical care in rural regions. This finding is supported by Karim et al [18], who found that online communities offer critical resources and spaces for gender affirmation. The digital world can allow individuals to create a sense of community and belonging even in the absence of physical proximity to others.

One crucial aspect of this study is the importance of health care providers having a foundational understanding of gender-affirming care, including knowledge of social and nonmedical aspects. Respect for proper pronouns and gender identities should be considered the bare minimum for creating inclusive and supportive environments. This may involve understanding the broader context of the struggles faced by gender-diverse people, especially in rural areas. Previous studies [19-21] have emphasized the need for health care systems to evolve to meet the needs of this community. Ignorance or neglect of these essential practices can further marginalize gender-diverse individuals, reinforcing the systemic barriers they face. By integrating these social and nonmedical aspects of care into standard practice, health care providers can contribute to a more inclusive and supportive environment for all gender-diverse individuals.

These findings illuminate the interplay between structural exclusion and community innovation—as formal health care systems remain inaccessible or invalidating, gender-diverse individuals generate alternative infrastructures of care through peer-to-peer support, online platforms, and social belonging.

### Limitations

Appropriate acknowledgment of limitations is crucial to inform the interpretation and application of the findings. One limitation included recruitment being conducted primarily through snowball sampling within community-based organizations. Thus, individuals who do not interact with these supports may not have known about the research. As most participants shared experiences from rural access standpoints in Nova Scotia, the findings may be less representative of urban-dwelling individuals. However, this geographic focus aligns with the acknowledgment that access to gender-affirming care services is more challenging in rural regions, thereby providing a unique strength in understanding the challenges faced.

The small sample size is a limitation; however, the depth of narratives, focused research question, and shared sociocultural context among participants provided substantial information power. While the findings are not intended to be generalizable, the sample allowed for a rich exploration of experiences within the Nova Scotia context.

Furthermore, this research did not explore how nuanced experiences of individuals with intersecting identities, such as race, ability, or culture, shed light on the complexities faced in accessing gender-affirming care. Additional research is needed to explore this intersection.

Finally, despite efforts to emphasize the inclusion of various types of gender-affirming care services on recruitment materials, some individuals may have associated gender-affirming care solely with traditional medical services, potentially leading to the exclusion of eligible participants. Moreover, gender-diverse persons who were not comfortable discussing their gender identity may not have chosen to participate; therefore, these voices may have been missed in the findings.

### Conclusions

These findings can inform health care providers’ perspectives on what services are important in affirming an individual’s gender identity and organizations that create policies to govern accessibility, including insurance companies and educational programs. It is recommended that policies prioritize the understanding and provision of gender-affirming care among health care providers, social service professionals, and community organizations in Nova Scotia. By conducting assessments to identify barriers to and facilitators of providing gender-affirming care, including medical and nonmedical interventions, targeted interventions, educational programs, and training initiatives can be developed to address gaps in knowledge, attitudes, and skills.

Future research directions should focus on exploring the perspectives of health care providers in nonmedical and informal gender-affirming care services to explore additional barriers to and/or facilitators of providing these services. Such work could aid in exploring how best to educate health care providers in gender-inclusive care and enable the best quality of care to be delivered to all individuals regardless of gender.
